# Housing insecurity, migration and HIV among sexual minority men in the U.S.

**DOI:** 10.1186/s13104-026-07712-x

**Published:** 2026-02-26

**Authors:** Susan Cassels, Sean C. Reid, Sofia Kaloper

**Affiliations:** Department of Geography, University of California Santa Barbara, Santa Barbara, CA 93106-4060 USA

**Keywords:** Housing insecurity, Sexual minority men, HIV, Migration, Social determinants of health, Health disparities, Spatial analysis

## Abstract

**Objective:**

Sexual minority individuals in the U.S. experience disproportionate levels of housing insecurity, shaped by structural inequalities, stigma and health related vulnerabilities. Drawing on minority stress theory and the sexual and gender minority health disparities framework, we investigate how housing insecurity among sexual minority men is associated with HIV status and domestic interstate migration, and how these relationships vary across space using data from four cycles of the American Men’s Internet Survey (2018–2021).

**Results:**

Among 42,401 sexual minority men in the study, a sample prevalence of 8.2% reported housing insecurity in the past year with significant state level variation. Pooled logistic regression models revealed HIV status was significantly associated with housing insecurity. Migrant status showed no independent association with housing insecurity after adjusting for education, as migrants were more likely to be highly educated, and more education was associated with lower risk of housing insecurity. Within the AMIS sample, respondents identifying as racial and ethnic minorities experienced elevated levels of housing insecurity. State-stratified models revealed geographic differences in the association between HIV status and housing insecurity, with the strongest effects observed in Utah, Colorado, Nevada, Texas, Georgia, Indiana and New Jersey, highlighting the critical need for tailored policies to address housing stability, particularly in geographic contexts represented within the AMIS sample.

## Introduction

Sexual minority individuals experience substantial health disparities, health risk factors, and unequal barriers to healthcare [1]. Additionally, the proportion of individuals who self-identify as lesbian, gay, bisexual or transgender in the U.S. has increased significantly over the past ten years, and is now at 9.3% [2]. Recent research has suggested that many fundamental needs, such as secure housing, are not being met among sexual minority men (SMM), especially SMM of color [3, 4]. Housing security is increasingly recognized as a priority for public health [5], as it is predictive of poor self-rated health among SMM [6] as well as a number of other health outcomes such as mental health and heart disease [7–9]. The mechanisms that link housing insecurity to poor health outcomes include delayed or postponing health care or medications [10], poor quality health care [10, 11], psychological changes, evictions, limited social support and stress [12], and physical exposures to poor housing quality [13]. Sexual minority populations experience housing insecurity at rates higher than heterosexual populations [14], but little research has looked into the patterns and drivers of housing insecurity among SMM [15]. We aim to examine how housing insecurity varies by demographic and health status over space for sexual minority men in the U.S., using data on HIV status and domestic interstate migration history.

We rely on the sexual and gender minority health disparities framework as well as minority stress theory to ground our research. A complex interplay of individual, interpersonal, community and societal factors impact the health and well-being of SMM. Additionally, minority stress theory argues that stigmatization leads to heightened stress levels among sexual and gender minority populations [16–19], suggesting a potential pathway from stigma to housing insecurity. Migration may be associated with less social capital, stress, and more sparse social networks [20], which could lead to housing insecurity as well. Additionally, SMM experience a disproportionately high burden of HIV in the U.S., accounting for more than 70% of new HIV diagnoses [21]. Living with HIV is associated with discrimination, stigma, and stress as well, and thus may also associated with instability and housing insecurity [22].

The severity and consequences of housing insecurity on poor health varies by space and place [17, 18]. Significant research on geographic and social determinants of health have argued that individual level characteristics and behaviors are often constrained or dictated by larger socio-ecological factors, and these can work together to create or maintain health disparities [23]. Thus, place is an important predictor of health [24], especially for SMM [25]. However, the spatial distribution of sexual minorities in the U.S. is still not clear given current national data sources. Knowing where SMM live is important for public health services and more [26–28]. A lack of representative data in national surveys has limited efforts to research spatial patterns of SMM populations in the United States [27, 29–32]. The U.S. census has only recently included questions about sexual orientation by asking about household relationship status, and the growing number of nationally representative surveys that include questions on sexual orientation and behavior are difficult to compare due to inconsistencies in survey question language. These limited data sets are also often restricted for privacy reasons, aggregated at low spatial resolution, and have limited temporal resolution. The lack of rich spatial and temporal data on the demographics of sexual minorities acts as a barrier for researchers, policy makers, and public health officials working to address disparities in this historically underserved population.

Housing insecurity is a significant social determinant of health, and the severity and consequences of housing insecurity varies by space and place. Our research aims to (1) describe spatial patterns of housing insecurity among sexual minority men in the U.S., (2) examine whether state-level migration status or HIV status are associated with housing insecurity. We hypothesize that housing security will be high and spatially patterned in the U.S. Additionally, we hypothesize that both HIV status and migration will be positively associated with housing insecurity. Migration may be associated with less social capital and more sparse social networks [20], which may lead to housing insecurity. People living with HIV may also have more housing insecurity, since living with HIV can cause instability.

## Data and methods

We use four cycles of the American Men’s Internet Survey (AMIS) from 2018 to 2021 (*n* = 42, 401) to examine spatial demography, health, and housing insecurity among SMM. AMIS is an anonymous cross-sectional online survey of HIV-related risk behaviors, testing, and use of prevention services among men who have sex with men in the United States. Complete surveys are available on the AMIS website (https://emoryamis.org/reports-and-surveys/). Informed consent to participate was obtained by all respondents, and the protocol was approved by the Emory University Institutional Review Board (#IRB00047676). The survey is conducted in annual cycles with a goal of at least 10,000 complete surveys each year. AMIS has occurred annually since 2013, and has recruited more than 100,000 surveys. In addition to a number of questions about HIV treatment and prevention, the online survey consists of a core questionnaire, including questions in the following domains: demographics, place of birth, current residence, foreign born status, housing insecurity and homelessness, mental health, stigma and social support.

Descriptive statistics, pooled bivariate and multivariate logistic regressions models with state-level fixed effects, as well as fully stratified logistic regression models by state, are used to examine associations between housing insecurity, migration, and HIV status. The descriptive and multivariate results are pooled across the multiple years of data as well. The key dependent variable is housing insecurity, measured as a respondent affirming that they doubled up or stayed overnight with friends, relatives, or someone they did not know well because they did not have a regular, adequate, and safe place to stay at night, during the past 12 months. Key independent variables include domestic interstate migration status, defined as being born in a different state than their current state of residence, and self-reported HIV status. The migration analysis is restricted to US born individuals.

We employ both pooled models with state fixed effects and fully stratified state-specific models to address complementary analytic goals. The pooled models estimate the average association between HIV status and housing insecurity across the AMIS sample while controlling for unobserved, time-invariant differences between states. Fully stratified models were chosen over state by HIV interaction terms to allow the association between HIV status and housing insecurity to vary flexibly across states and to produce directly interpretable state-specific estimates and confidence intervals, without imposing parametric assumptions about how effects differ by state. This approach improves geographic specificity and highlights spatial heterogeneity in associations, but comes at the cost of reduced precision in states with smaller sample sizes.

## Results

Table 1 describes key variables from the AMIS data, which is limited to state-level geographies and years 2018–2021. A total of 42,401 self-identified sexual minority men comprise our sample. Among the pooled sample, a little more than 8% reported that they had experienced housing insecurity in the last 12 months. More than a third of respondents over the 4 waves (38%) were migrants, or born in a different state than their current state of residence. About 12% were non-Hispanic Black, 17% Hispanic, 64% non-Hispanic white, and 8% other or multiple races. The average education level among respondents was high, with more than 95% of the sample having a high school diploma or higher. Respondents’ ages ranged from 15 to 92 years, with a median of 29 years, and mean age of 35 years.

**Table 1:**
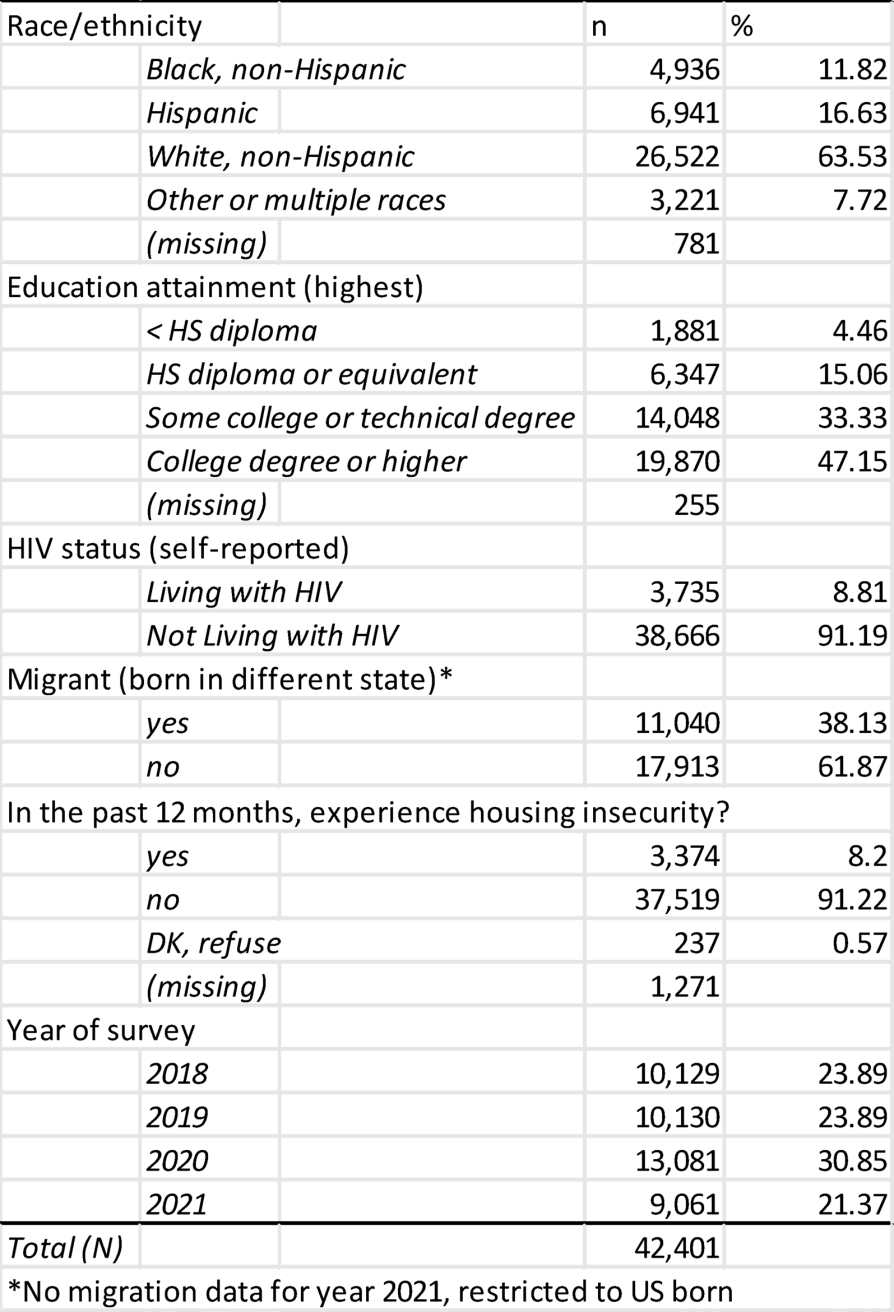
Socio-demographic and housing characteristics of the AMIS sample, years 2018–2021

**Fig. 1:**
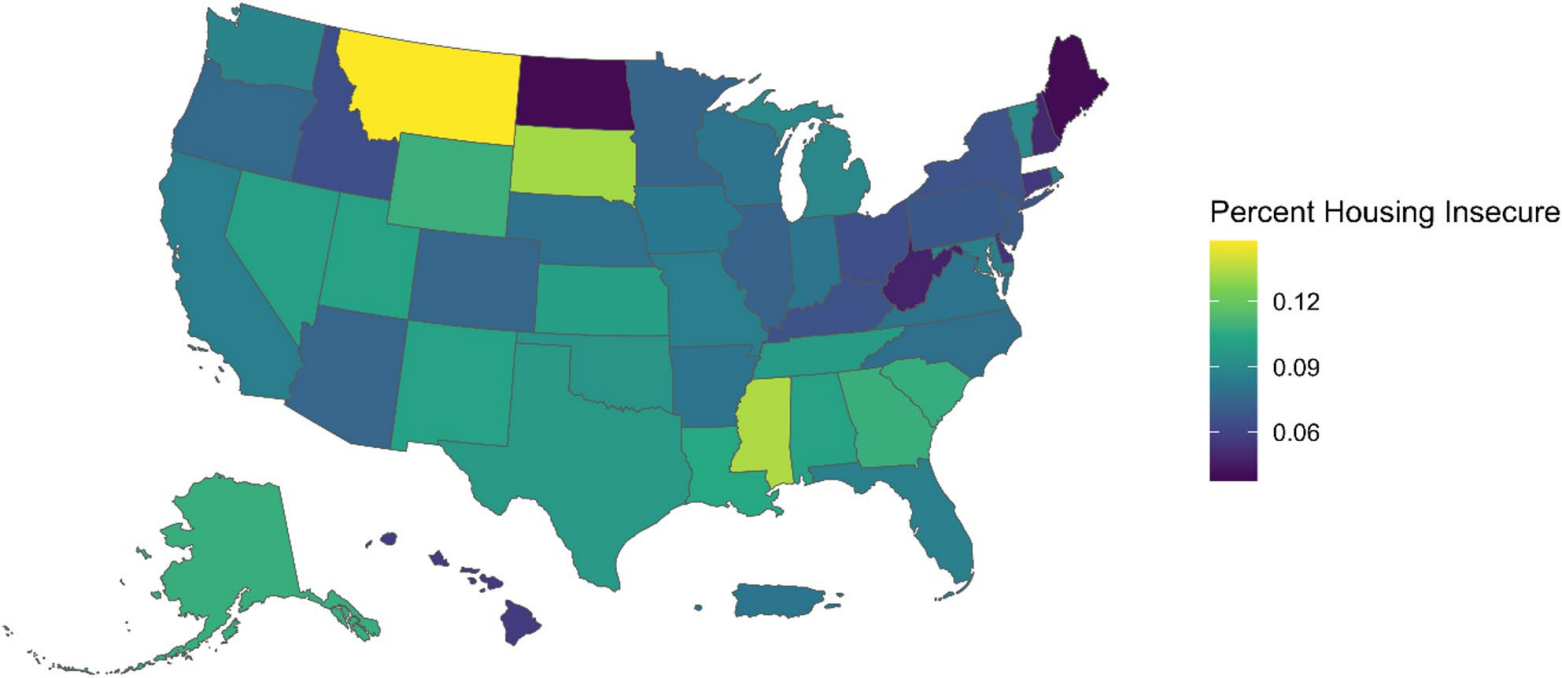
Sample prevalence of housing insecurity by state, among sexual minority men who responded to the AMIS survey, 2018–2021

Rates of housing insecurity vary across space among participants in the sample. The average rate of housing insecurity was 8.2%, with state-level estimates ranging from approximately 3.7% − 14.8%. Figure 1 displays variation in reported housing insecurity by state among AMIS respondents, with lower reported rates in states such as North Dakota and Maine and higher reported rates Montana and several states in the South, central plains, and Mountain West.

Sample prevalence of HIV and domestic interstate migration vary spatially as well (data not shown). Higher proportions of AMIS respondents who are interstate migrants are generally seen along the east and west coasts. States with higher proportions of AMIS respondents who are living with HIV are also found along both coasts, as well as the South.

Results of the pooled bivariate logistic regressions models suggest a negative and significant association between housing insecurity and migration status [OR = 0.77] but a positive and significant association between housing insecurity and HIV status [OR = 1.42] (results not shown). However, once we control for additional variables, including age of respondent, race and ethnicity, and education, the negative association between migration status and housing insecurity goes away. Specifically, migrant SMM were significantly more likely to be college-educated, and higher education was associated with less housing insecurity. Thus, education is a significant confounder. The association between HIV status and housing insecurity continues to be positive statistically significant [OR = 1.7, 96%CI: 1.45–2.02]. Similar to other research on housing insecurity, racially and ethnically minorities individuals, as well as individuals with less education, have higher levels of housing insecurity (see Table 2).

**Table 2:**
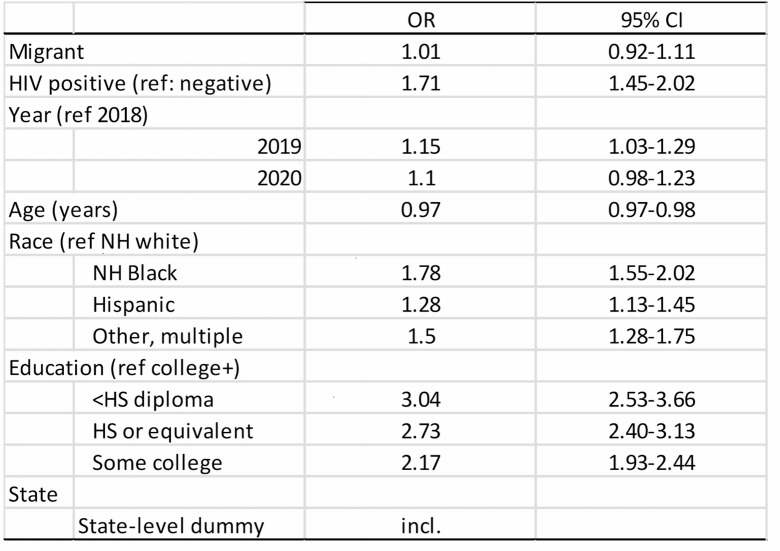
Pooled logistic regression model (AMIS data from 2018, 2019, 2020), to examine factors associated with housing insecurity among sexual minority men in the U.S. (the migration variable is unavailable for the year 2021)

**Fig. 2:**
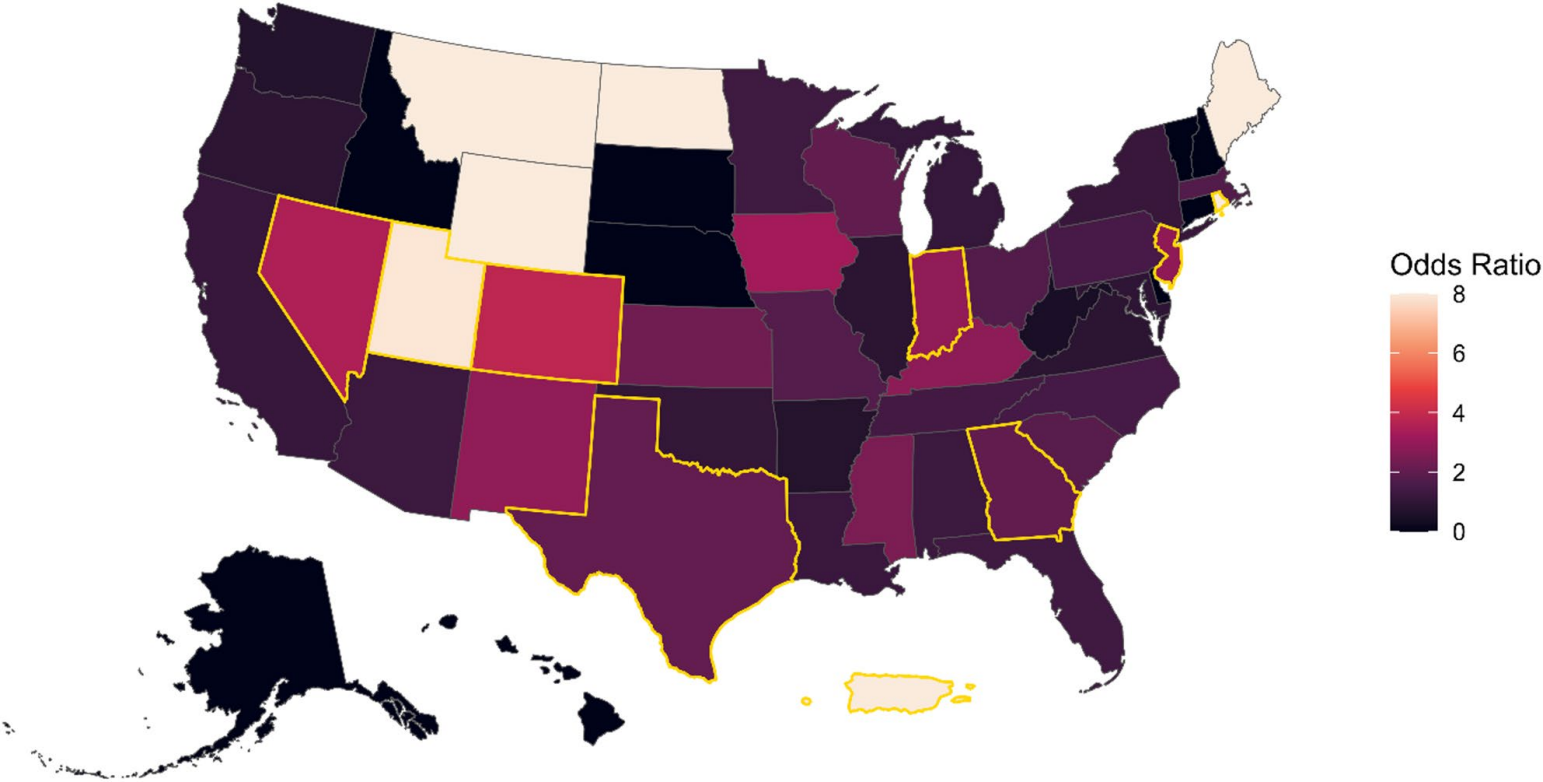
State-specific multivariate logistic regressions model, depicting the association (odds ratios) between HIV status and housing insecurity. Model controls for year, age, education and race & ethnicity. Insufficient sample size for states in white. Outline in gold represents statistical significance

Lastly, since both housing insecurity and HIV status varied significantly by state, we ran stratified multivariate logistic models by state, to assess whether the positive association between HIV status and housing insecurity also varied across space. Model specifications were the same as the model in Table 2, but without controlling for migration status. Four state-specific models with small sample sizes did not converge, (Maine, Wyoming, North Dakota and Montana). The odds of experiencing housing insecurity among those living with HIV were greater than one for all but six states, (Washington, Oregon, Maryland, Illinois, Arkansas and Virginia). Figure 2 displays the adjust odds ratios for the multivariate logistic regression models by state, with the color representing the odds ratios and the shade representing the confidence in the association. States with the strongest positive association between HIV status and housing insecurity included Utah [OR = 7.88, 95%CI: 2.4–25.5], Colorado [OR = 3.8, 95%CI: 1.5–9.9], Nevada [OR = 3.4, 95%C: 1.1–10.7], Indiana [OR = 2.9, 95%CI: 1.1–7.1], New Jersey [OR = 2.89, 95%CI: 1.1–7.6], Texas [OR = 2.05, 95%CI: 1.4–2.9], and Georgia [OR = 2.01, 95%CI: 1.4-3.0]. Again, as these are fully stratified models by state, some samples sizes are small and thus confidence intervals are wide. There does not appear to be a geographic pattern to these state-specific results. Nonetheless, these results should be viewed as descriptive of the AMIS sample, and interpreted with caution.

## Discussion

Our results demonstrate that the sample prevalence of housing insecurity is high among SMM who participated in the AMIS survey and that sample prevalence of housing insecurity varies significantly by state. Contrary to our hypotheses, migrant SMM, or those who are living in a state different than their state of birth, were less likely to experience housing insecurity. However, this relationship was no longer significant once we accounted for education. We treated education as a confounder in the model, as education was associated with migration (migrant men were much more likely to have a college degree, at 53% compared to 35% for non-migrant men), and education was associated with housing insecurity (those with higher education were less likely to experience housing insecurity). However, we are unable to assess whether acquiring higher education directly leads to lower risk of housing insecurity. Additionally, our measure of domestic interstate migration is quite coarse, as it cannot account for the timing, duration, or reason for migration. Therefore, our null association may mask heterogeneous associations between subgroups of migrants.

On the other hand, HIV status was strongly associated with housing insecurity, with people living with HIV experiencing housing insecurity at rates 70% higher than those not living with HIV. HIV status was positively associated with housing insecurity in most state-specific models estimated from the AMIS sample, but the strength and magnitude of the association varied across space. Some of the strongest positive associations between housing insecurity and HIV status were found in Utah, Colorado, Nevada, Texas, Georgia, Indiana and New Jersey, within the AMIS data. Interestingly, there does not appear to be a geographic pattern to these associations. Although not statistically significant, a number of states displayed a negative association between HIV status and housing insecurity (i.e., odds ratios less than one), including Washington, Oregon, Maryland, Illinois, Arkansas and Virginia. Some of these states are known for their progressive and strong HIV-related public health efforts, such as Washington State. Future work should examine whether HIV-related policies or larger social and structural level drivers moderate the associations between HIV status and housing insecurity.

## Conclusion

Within the AMIS sample, HIV status was strongly associated with higher odds of housing insecurity, and the strength of this association varied across states. The causal mechanisms are unclear, as both unstable living conditions can lead to higher HIV risk, stigma and stress, but also HIV diagnoses are highly associated with subsequent homelessness or housing instability. As theorized by minority stress theory and SGM health disparities framework, SMM living with HIV may lose housing due to such factors as stigma and discrimination, increased medical costs and limited incomes, or reduced ability to keep working due to HIV-related illnesses, or housing insecurity may lead to higher risk of HIV acquisition.

### Limitations

This study relies on self-reported data reported in the American Men’s Internet Survey which may be subject to recall bias or social desirability bias, particularly for sensitive topics like housing insecurity and HIV status. The survey’s online recruitment may underrepresent sexual minority men with limited internet access or those actively experiencing severe housing instability, which may skew the sample towards more socioeconomically advantaged individuals. Additionally, our measure of housing security specifically mentions “doubling up or staying overnight with others,” which only captures a narrow dimension of insecure housing. Our measure captures episodic housing instability, and thus may be a conservative estimate base on broader definitions of insecurity. Nonetheless, this was the most consistent indicator available across the AMIS survey data. Furthermore, this analysis is aggregated at the state level, which may mask finer-scale spatial variations in housing insecurity and HIV prevalence within states. Small sample sizes in some states led to non-converging models, reducing the robustness of state-specific findings. Our analysis pooled multiple years of data as well, spanning the COVID pandemic. Issues around housing security were likely exacerbated by the pandemic, but we did not isolate pandemic-specific effects. Future research should aim to analyze trends of housing insecurity at finer spatial scales to account for any biases introduced by the aggregation, as well as over time to identify whether there were temporal trends in HIV, housing and migration, or to identify COVID-specific effects.

## Data Availability

The data that support the findings of this study are available from PRISM Health at Emory University but restrictions apply to the availability of these data, which were used under license for the current study, and so are not publicly available. Data may be available upon reasonable request and with permission of PRISM Health at Emory University.

## Abbreviations

SMM: Sexual minority men
SGM: Sexual and gender minority
AMIS: American men’s internet survey
HIV: Human immunodeficiency virus
OR: Odds ratio
CI: Confidence interval
